# Planning target volume margins for prostate radiotherapy using daily electronic portal imaging and implanted fiducial markers

**DOI:** 10.1186/1748-717X-5-52

**Published:** 2010-06-10

**Authors:** David Skarsgard, Pat Cadman, Ali El-Gayed, Robert Pearcey, Patricia Tai, Nadeem Pervez, Jackson Wu

**Affiliations:** 1 Department of Radiation Oncology, Tom Baker Cancer Center and University of Calgary; 1331 29 St NW, Calgary AB, T2N 4N2, Canada; 2 Department of Medical Physics, Saskatoon Cancer Center; 20 Campus Drive, Saskatoon SK, S7N 4H4, Canada; 3 Department of Radiation Oncology, Saskatoon Cancer Center; 20 Campus Drive, Saskatoon SK, S7N 4H4, Canada; 4 Department of Radiation Oncology, Cross Cancer Institute; 11560 University Ave, Edmonton AB, T6G 1Z2, Canada; 5 Department of Radiation Oncology, Allan Blair Cancer Center; 4101 Dewdney Avenue, Regina SK, S4T 7T1, Canada

## Abstract

**Background:**

Fiducial markers and daily electronic portal imaging (EPI) can reduce the risk of geographic miss in prostate cancer radiotherapy. The purpose of this study was to estimate CTV to PTV margin requirements, without and with the use of this image guidance strategy.

**Methods:**

46 patients underwent placement of 3 radio-opaque fiducial markers prior to prostate RT. Daily pre-treatment EPIs were taken, and isocenter placement errors were corrected if they were ≥ 3 mm along the left-right or superior-inferior axes, and/or ≥ 2 mm along the anterior-posterior axis. During-treatment EPIs were then obtained to estimate intra-fraction motion.

**Results:**

Without image guidance, margins of 0.57 cm, 0.79 cm and 0.77 cm, along the left-right, superior-inferior and anterior-posterior axes respectively, are required to give 95% probability of complete CTV coverage each day. With the above image guidance strategy, these margins can be reduced to 0.36 cm, 0.37 cm and 0.37 cm respectively. Correction of all isocenter placement errors, regardless of size, would permit minimal additional reduction in margins.

**Conclusions:**

Image guidance, using implanted fiducial markers and daily EPI, permits the use of narrower PTV margins without compromising coverage of the target, in the radiotherapy of prostate cancer.

## Background

Several randomized trials have shown improved biochemical relapse-free survival with the use of higher doses of radiotherapy (RT) in subsets of patients with organ-confined prostate cancer [1-3]. Although such higher doses may result in a greater risk of acute and late toxicity [4], these risks may be mitigated by the use of narrower normal tissue margins around the target. Narrower margins could, however, lead to an increased risk of geographic miss, because of variation in the day-to-day position of the prostate relative to the skin markings (inter-fraction motion), and internal movement of the prostate over the course of a single treatment (intra-fraction motion).

In order to reduce the risk of geographic miss, radio-opaque fiducial markers can be implanted within the prostate. Electronic portal imaging (EPI) is then performed prior to each treatment, and isocenter placement errors are corrected if they exceed pre-determined tolerance levels [5]. This approach minimizes the effect of systematic and random set-up error, such that the ultimate accuracy of the treatment should depend solely on residual error inherent to the correction protocol that is used, together with intra-fraction motion of the target.

A prospective phase I/II study was conducted at four regional cancer centers in the Canadian provinces of Alberta and Saskatchewan, to evaluate acute and late toxicity associated with the use of a hypofractionated RT schedule of 55 Gy in 16 fractions over four weeks (4 fractions/week), using image guidance with fiducial markers and daily EPIs. The purpose of this study was to examine the size of PTV margins that would be required to confidently cover the target, without and with the use of the above image guidance strategy.

## Methods

### Patient data

A total of 72 patients were recruited to a prospective multicenter phase I/II trial between 2004 and 2006 of escalated biological dose short course hypofractionated radiotherapy for low and intermediate risk prostate cancer. Eligible patients had to have low or intermediate risk adenocarcinoma, stage T1-T2b N0-x M0, with a Gleason score of 7 or less and a PSA level of not more than 20. Patients were ineligible if they had a prosthetic hip or other similar hardware that would interfere with visualization of the fiducial markers on daily portal images. The study was approved by the local Research Ethics Board of each participating institution, and all patients signed a study-specific consent form.

This report describes positioning and targeting accuracy in the 46 patients on this study who were treated on conventional linear accelerators without integrated couch adjustment systems. A further 26 patients who were treated on a dedicated stereotactic unit with on-board kV imager and an integrated couch adjustment system were excluded from the present analysis.

### Preparation and treatment planning

All patients underwent implantation into the prostate of 3 gold marker seeds (24 K gold, 0.95 mm in diameter and 3 mm in length) under trans-rectal ultrasound guidance. The gold seeds were placed in the prostate base, mid-gland and apex. Antibiotic prophylaxis was used, and typically consisted of ciprofloxacin 500 mg twice daily for three days, starting the day before the implantation procedure.

Patients then underwent CT-simulation in the supine position, with immobilization according to the institutional standard. This typically consisted of a non-customized foot holding device, in some cases with the addition of a soft roll behind the knees. Rigid immobilization devices were not used. Patients were instructed to have a filled bladder and an empty rectum for their CT-simulation and for each treatment appointment. Bowel and bladder instructions that were given to patients were institution specific but typically involved the ingestion of a specified amount of water at a certain interval prior to treatment, and the use of a mild laxative such as milk of magnesia as needed to maintain a regular bowel habit. A suppository or enema prior to CT-simulation was recommended but was not mandatory. The CT simulation was performed without contrast, at a slice thickness of 3 mm. Urethrograms were not performed.

The clinical target volume (CTV) consisted of the prostate gland +/- the proximal seminal vesicles. The planning target volume (PTV) was created by symmetrically expanding the CTV by 1.0 cm in all directions except posteriorly, where it was expanded by 0.5 cm. This was done empirically because of uncertainty about rectal toxicity with this hypofractionated RT regimen, and we anticipated there would be reliable coverage of the CTV with the use of daily image guidance.

Patients were planned and treated in the supine position using 3-dimensional conformal RT (3D-CRT) or, if dose constraints of the study could not be met, with intensity modulated RT (IMRT). The prescription dose was 55 Gy in 16 fractions over 4 weeks, delivered as 4 fractions per week. The PTV was required to be covered by 98% of the prescription dose and none of the CTV was allowed to receive less than 55 Gy.

High resolution digitally reconstructed radiographs (DRRs) were generated for the anterior (0°) and lateral (90° or 270°) gantry angles, whether or not they were actual treatment fields, and these were electronically attached to the patient's file in the Varis Vision^® ^system.

### Target localization and treatment delivery

Patients were positioned each day for radiotherapy by lining up room-mounted lasers to skin markings that had been made at the time of CT-simulation, then making a prescribed set of moves as dictated by the treatment plan to arrive at a skin entry point that was consistent across all treatments. This was the standard practice at the time of the study at all 4 participating institutions, for prostate patients who were being treated without image guidance.

Daily orthogonal electronic portal images (EPIs) were then taken from the anterior and lateral gantry angles, from a consistent skin entry point as defined above. A total of 32 images were planned (16 anterior, 16 lateral) for each treatment course. A radiation dose of 8 monitor units (approximately 4 -- 6 cGy at the prescription point) was attributed to each image, and this dose was incorporated into the treatment plan such that the total delivered dose remained at 5500 cGy.

The position of the gold markers on each daily pair of EPIs was compared to their intended position, as seen on the reference DRR, to determine isocenter placement error, by using the anatomy matching functions of the Varis Vision^® ^software. The anterior EPI was used to determine error along the left-right (L-R) axis, while the lateral EPI was used to determine error along the superior-inferior (S-I) and anterior-posterior (A-P) axes.

Tolerance for isocenter placement error was empirically defined as less than 3 mm along the L-R and S-I axes, and less than 2 mm along the A-P axis. Therefore, if an isocenter placement error of 3 mm or greater was measured on any treatment day along the L-R and/or S-I axes, the lateral and/or longitudinal position of the treatment table was adjusted as needed to completely correct this error. Similarly, if an isocenter placement error of 2 mm or greater was measured along the A-P axis, the table height was adjusted as needed to completely correct this error. At all participating institutions, this required radiation therapy staff to enter the treatment room and manually adjust the couch position in the opposite direction to the error along each of the affected axes. Rotation could be used, if necessary, to facilitate matching, but rotational errors were not recorded or corrected. Localization EPIs were not repeated to confirm that isocenter placement errors had been corrected properly prior to treatment, because the additional dose of radiation that would have been incurred by this *ad hoc *procedure had not been accounted for in the planning process.

Repeat EPIs were captured during treatment delivery, again from anterior and lateral gantry angles. Although the protocol did not specify when these were to be done, they were typically performed about mid-way through the treatment fraction. With the use of an amorphous silicon electronic portal imaging device at the high resolution setting and at the appropriate photon energy, the gold seeds were well visualized in all of our patients. The position of the isocenter on these verification EPIs was compared with its intended position as per the DRRs, along the L-R, S-I and A-P axes. Since the isocenter position on the during-treatment EPIs could have been affected by both intrafraction motion and residual uncorrected isocenter placement error, we used the following formula to estimate the magnitude of intrafraction motion alone:

where L-R_2 _, S-I_2 _and A-P_2 _, and L-R_1 _, S-I_1 _and A-P_1_, represent during-treatment and pre-treatment (uncorrected) isocenter positions along the L-R, S-I and A-P axes respectively, and c_L-R _, c_S-I _and c_A-P _represent the corrections that were made along each of those axes. For example, if the pre-treatment (uncorrected) isocenter position along the S-I axis was +4 mm, such that a correction of -4 mm was made before treatment, and the during-treatment isocenter position was -2 mm, then the estimated intra-fraction motion along the S-I axis would be (-2) -- (+4) -- (-4) = -2 mm. PTV margins that would be required to give 95% probability of CTV coverage on any treatment day were calculated using the method described by Antolak [6]. Briefly, this involved expanding the CTV in three dimensions using an ellipsoid with major axes of 1.65 times the total standard deviation in each direction.

## Results

Table 1 shows the clinical characteristics of the 46 patients included in the study.

**Table 1:** Patient characteristics (n = 46)

Age (years)	
Median	70
Mean	68.6
SD	6.9
T-category (%)	
T1a	1 (1%)
T1c	20 (43%)
T2a	11 (24%)
T2b	8 (17%)
T2c	5 (11%)
Unknown	1 (2%)

Gleason score (%)	
3 + 3	20 (43%)
3 + 4	17 (37%)
4 + 3	9 (20%)

No. of positive biopsy cores (%)	
2 or fewer	15 (33%)
3 - 4	16 (35%)
5 or more	12 (26%)
Unknown	3 (7%)

Last pre-treatment PSA (%)	
0 - 3.9	8 (17%)
4.0 - 9.9	29 (63%)
10.0 - 14.9	7 (15%)
15.0 - 20.0	2 (4%)
Mean	6.8
Median	6.3
Minimum	0.4
Maximum	19.4

### Isocenter placement accuracy with set-up relative to skin markings

**Figure1:**
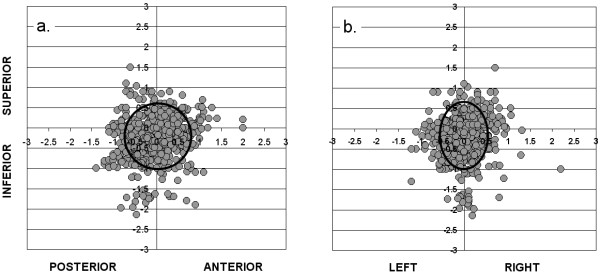
**Isocenter placement errors (in cm) relative to DRR on pre-treatment EPIs (gray circles; n = 736 fractions), along a): S-I and A-P axes, and b): S-I and L-R axes**. Ellipse shows 95% confidence intervals for CTV coverage in each direction.

Figure 1 shows, for each fraction of RT that was given, the isocenter placement error on the pre-treatment EPIs, relative to the intended position of the isocenter on the corresponding reference image, along the S-I and A-P (Figure 1a) and the S-I and L-R (Figure 1b) axes. Summary statistics are shown in Table 2, in the left-hand columns ("pretreatment"). These EPIs were taken after the patient had been set up for treatment in the conventional fashion using the previously-described method. Any deviation of individual points from the intersection of the x and y axes represents the isocenter placement error for one fraction. The mean pre-treatment isocenter position (± SD), relative to that on the reference image was 0.01 ± 0.35 cm, -0.24 ± 0.48 cm and 0.01 ± 0.47 cm along the L-R, S-I and A-P axes respectively. As these numbers indicate, although confidence intervals overlap zero, there was a trend toward a systematic error of over 2 mm in the inferior direction, which may be due to greater patient relaxation during treatment than at the time of CT-simulation. The ellipse on each of figures 1a and 1b indicates the 95% confidence interval for isocenter placement along each axis, relative to the reference image. If daily set-up verification and correction were not performed, CTV to PTV margins of 0.57 cm, 0.79 cm and 0.77 cm would be required along the L-R, S-I and A-P axes respectively, to give a 95% probability of complete CTV coverage on any given treatment day.

**Table 2:** Pre-treatment and during treatment isocenter placement errors

	Pre-treatment (cm)					During treatment (cm)				
	**Min**	**Mean**	**Median**	**Max**	**SD**	**Min**	**Mean**	**Median**	**Max**	**SD**

A-P mismatch	-1.40	0.01	0.03	2.00	0.47	-1.10	0.03	0.03	0.75	0.22

R-L mismatch	-1.20	0.01	0.00	2.20	0.35	-2.66	0.01	0.02	0.80	0.22

S-I mismatch	-2.15	-0.24	-0.20	1.50	0.48	-0.89	0.01	0.00	1.15	0.22

Of the total 736 daily fractions that were administered, pre-treatment EPIs showed isocenter placement errors that exceeded protocol specifications (3 mm or more in all directions except 2 mm or more along the anterior-posterior axis) in 31%, 52% and 63% of treatments along the L-R, S-I and A-P axes respectively, of which 14%, 31% and 29% were larger than 5 mm. In 88% of all treatments, the patient's position had to be adjusted because of an isocenter placement error that exceeded tolerance limits along one or more axes. In 55% of all treatments, the initial set-up without image guidance resulted in an isocenter placement error of greater than 5 mm along at least 1 axis.

### Isocenter placement accuracy during-treatment, using a daily EPI and correction protocol

**Figure 2:**
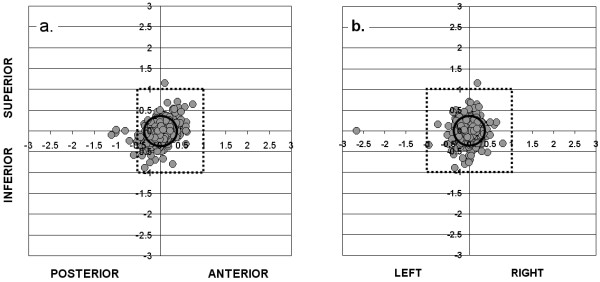
**Isocenter placement errors (in cm) relative to DRR on during-treatment EPIs (gray circles; n = 530 fractions), along a): S-I and A-P axes, and b): S-I and L-R axes**. Outer box shows PTV margins used in the study; inner ellipse shows 95% confidence intervals for CTV coverage in each direction.

Figure 2 shows, for each fraction of RT, the during-treatment isocenter position relative to its intended position on the reference image along the S-I and A-P (Figure 2a) and the S-I and L-R (Figure 2b) axes. Summary statistics are shown in Table 2, in the right-hand columns ("during treatment"). In the figures, any deviation of individual points from the intersection of the x and y axes represents a combination of residual (uncorrected) pre-treatment isocenter placement error (i.e. within the tolerance limits of the correction protocol) and intra-fraction motion. The mean during-treatment isocenter position (± SD), relative to that on the reference image, was 0.01 ± 0.22 cm, 0.01 ± 0.22 cm and 0.03 ± 0.22 cm along the L-R, S-I and A-P axes respectively. As these numbers indicate, after correction of pre-treatment errors according to our protocol, there was no significant remaining systematic error in position of the isocenter compared to the reference images.

The inner ellipse on each of figures 2a and 2b indicates the 95% confidence interval for isocenter placement relative to the reference image. With our correction protocol, CTV to PTV margins of 0.36 cm, 0.37 cm and 0.37 cm would be required along the L-R, S-I andA-P axes respectively, to give a 95% probability of complete CTV coverage on a given treatment day. The percentage of treatments having an isocenter placement error of 5 mm or greater in any direction on the during-treatment EPIs was 8.3%. The outer box on each of these figures shows the PTV margins that were used on this protocol; 10 mm in all directions except posteriorly, where a 5 mm margin was used. As can be seen, these margins gave adequate coverage of the CTV in almost all of the 530 fractions for which during-treatment EPIs were taken. In one case, a 2.7 cm isocenter placement error on the during-treatment EPI was observed. This was attributed to a mistake that was made on the treatment unit in correcting a 3 mm error along the L-R axis on the pre-treatment EPI (figure 2b). Although this point was included in our calculation of CTV to PTV margins required for 95% probability of CTV coverage, the exclusion of this one point would have had little effect on the result.

### Intra-fraction motion

**Figure 3:**
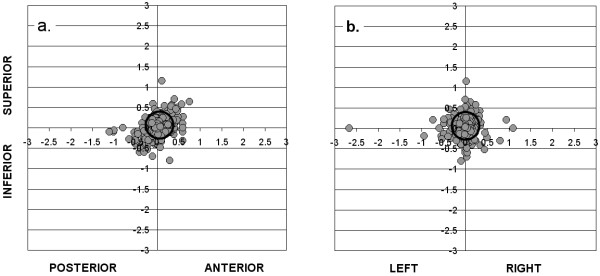
**Isocenter placement errors (in cm) on during-treatment EPIs (gray circles; n = 530 fractions), relative to the expected pre-treatment isocenter position, along a): S-I and A-P axes, and b): S-I and L-R axes**. Ellipse shows 95% confidence intervals for CTV coverage in each direction.

Figure 3 shows, for each fraction of RT, the estimated intra-fraction motion (assuming there was no residual uncorrected isocenter placement error prior to treatment), along the S-I and A-P axes (figure 3a) and the S-I and L-R axes (figure 3b). The mean intrafraction motion (± SD) was 0.01 ± 0.20 cm, 0.05 ± 0.19 cm and 0.04 ± 0.21 cm along the L-R, S-I and A-P axes respectively. Because the means are close to zero along each axis, this suggests that intra-fraction motion was a random process in the population of patients that we studied.

The ellipse on figures 3a and 3b indicates the 95% confidence interval for isocenter placement relative to its pre-treatment position, which was assumed to be the intended isocenter position. If all pre-treatment isocenter placement errors were completely corrected, regardless of size, leaving intra-fraction motion as the only variable affecting during-treatment isocenter placement, PTV margins of 0.33 cm, 0.32 cm and 0.35 cm would be required along the L-R, S-I and A-P axes respectively, to give a 95% probability of complete CTV coverage on any given treatment day.

## Discussion

The use of implanted fiducial markers, with daily pre-treatment electronic portal imaging during a course of prostate RT, makes it possible to estimate the extent of variation in prostate position relative to external skin markings, from one fraction to another (inter-fraction motion), and during a single fraction (intra-fraction motion). We found that the use of daily image guidance by fiducial markers and a threshold-based correction process would have permitted a substantial reduction in PTV margins, from 0.57 cm, 0.79 cm and 0.77 cm to 0.36 cm, 0.37 cm and 0.37 cm in the left-right, superior-inferior, and anterior-posterior directions respectively. Our strategy of adjusting the patient's position if necessary prior to treatment, to correct isocenter placement errors of 3 mm or larger along the L-R and S-I axes, and 2 mm or larger along the A-P axis, effectively reduced the combined systematic and random error to within 3 mm along the L-R and S-I axes and 2 mm along the A-P axis.

Our image guidance procedure of taking a pre-treatment EPI, comparing it to the reference image and adjusting the patient position as required, added about 5 minutes to the daily treatment time. On this protocol with only 16 fractions per treatment course, this extra time on the treatment machine was more than made up for by the reduction in number of fractions compared to conventional regimens of 35 to 39 fractions.

We wondered whether correction of all isocenter placement errors on the pre-treatment EPIs, regardless of size, would have permitted the use of even narrower CTV to PTV margins than are shown in figures 2a and 2b. To estimate the CTV to PTV margins that would be required to account only for intra-fraction motion, assuming there was no residual (uncorrected) isocenter placement error, we re-constructed figures 2a and 2b after normalizing the pre-treatment position to zero along each axis, to mimic the situation in which all isocenter placement errors are corrected. By comparing CTV to PTV margins in figures 3a and 3b with those in figures 2a and 2b, it can be seen that residual (uncorrected) isocenter placement error plays a very small role, compared to intra-fraction movement, in determining the ultimate accuracy of treatment. We estimated that, had we corrected all isocenter placement errors along each of the 3 axes, we would have been able to further reduce CTV to PTV margins by not more than 0.05 cm along any axis, and by a clinically meaningless 0.02 cm along the most significant A-P axis. This indicates that, at least on treatment machines with non-automated correction of isocenter placement errors, there is little to be gained from correcting errors that are smaller than the tolerance levels that were used in this study. Automated, operator-independent correction of all isocenter placement errors would, however, remove the risk of human error that resulted, for example, in a 2.7 cm error in the "corrected" isocenter position, as shown in Figure 2b.

Table 3 shows intra-fraction motion (IFM) estimates from a selection of published reports. A variety of different methods have been used to estimate IFM, including i) fiducial markers imaged with EPID and/or port films [present study, 7 -- 10], cone beam CT [11] and aSi "movies" [12]; ii) real-time monitoring of the position of electromagnetic transponders [13]; iii) cine-MRI [14]; iv) B-mode acquisition and targeting (BAT) ultrasound [15]; and v) serial CT scans [16].

**Table 3:** Intra-fraction motion (IFM) in various series

Series (no. of patients)	Treatment set-up details	Standard deviation of IFM (cm)			Comments
			
		L - R	S - I	A - P	
Present series (n = 46)	Supine, knee cushion. Comfortably full bladder, empty rectum.	0.20	0.19	0.21	3 fiducial markers, imaged with aSi EPID. IFM estimated by comparing during-treatment EPI isocenter position with presumed pre-treatment position (after any correction; not verified by a repeat EPI).

Cheung [7] (n = 33)	Custom vacuum lock bag. Empty bladder and rectum.	0.09	0.12	0.18	3 fiducial markers, imaged with EPID. IFM estimated by comparing pre and post-treatment EPIs on days 1 to 9 of phase I.

Aubry [8] (n = 18)	Supine, immobilization not stated. Full bladder, empty rectum.	0.08	0.11	0.16	2 - 3 implanted fiducial markers. Multiple daily sets of portal images to estimate intrafraction motion. IFM was < 5 mm in 100%, 99.5% and 99% of cases along L - R, S - I and A - P axes respectively.

Chung [9] (n = 17)	Supine, custom vacuum lock bag, standard leg immobilizing device. Comfortably full bladder, empty rectum.	ns	0.25	0.32	3 implanted fiducial markers. Lateral portal images prior to treatment. Correction of isocenter placement errors > 3 mm in any direction. Post-correction EPI to confirm correction.

J Wu [10] (n = 13)	Supine, alpha cradle, soft foam immobilization device supporting lower legs. Partially full bladder, empty rectum.	ns	0.21	0.23	3 implanted fiducial markers. Daily EPI to confirm field placement. 3 × weekly lateral port films to measure random and systematic field placement errors. Data shown are with respect to center of mass.

Letourneau [11] (n = 8)	Not stated	ns	0.09	0.09	3 implanted fiducial markers. Initial set-up according to skin marks, then cone beam CT verification of marker position and correction as required, followed by repeat cone beam CT for confirmation. Movement of markers relative to bony landmarks was assessed with kV x-rays; shown are standard deviations of IFM based on first and last radiographs that were taken between the 2 cone beam CTs, approximately 15 - 25 minutes apart.

Nederveen [12] (n = 10)	Supine, knee cushion. Empty bladder; no bowel instructions.	ns	0.07	0.05	Real-time aSi "movies" showing movement of fiducial markers within the prostate over a 2 - 3 minute period.

Litzenberg [13] (n = 11)	Supine, flat couch, knee support. No bladder or bowel instructions.	0.02	0.12	0.08	3 electromagnetic transponders (Calypso^®^) implanted in the prostate. Monitoring of position of transponders for 8 minutes.

Ghilezan [14] (n = 6)	Supine, no immobilization. Empty bladder, full rectum.	ns	0.17 (mid-posterior) 0.13 (apex)		Sagittal cine-MRI at 6 sec intervals over 1 hour on 3 days. Measured movement was in sagittal plane; no distinction between A - P and S - I axes. Rectal filling based on qualitative assessment of the amount of gas and feces in the rectum on a particular scan.
		
	As above, empty rectum.	ns	0.08 (mid-posterior) 0.10 (apex)		

Huang [15] (n = 20)	Supine. No additional details.	0.04	0.10	0.13	BAT ultrasound images before and after treatment. IFM was < 5 mm in 100%, 99.5% and 99% of cases along L - R, S - I and A - P axes respectively.

Stroom [16] (n = 15) a) Supine	Supine, knee roll, foot support. Suppository prior to planning CT; partially full bladder for all CTs.	0.06	0.25	0.28	Planning CT, 3 repeat CTs, at 2, 4 and 6 weeks of treatment. Changes in CTV position relative to bony anatomy were compared on the 4 CT datasets to estimate IFM.

Stroom [16] (n = 15) b) Prone	Prone with belly board. Otherwise as above.	0.05	0.15	0.17	As above.

*Abbreviations*: L - R = left to right; S - I = superior to inferior; A - P = anterior to posterior; aSi = amorphous silicon; EPID = electronic portal imaging device; EPI = electronic portal image; ns = not stated; BAT: B-mode acquisition and targeting.

**Table 4:** CTV to PTV margin recommendations in various series, without image guidance

Series (number of patients)	Treatment set-up details	CTV - PTV margin requirement (cm)			Comments
			
		L - R	S - I	A - P	
Present series (n = 46)	Supine, knee cushion. Comfortably full bladder, empty rectum.	0.57	0.79	0.77	3 fiducial markers, no correction of isocenter placement errors. Margins required for 95% probability of CTV coverage for any given fraction.

van der Heide [5] (n = 453)	Supine, knee cushion. Empty bladder, no bowel instructions.	0.36	0.48	0.79	2 - 4 fiducial markers. Daily aSi EPI. Results without application of a correction protocol. Standard deviations were provided, from which we calculated margins required to give 95% probability of CTV coverage (CTV - PTV margin calculated as SD × 1.65 [6]).

Litzenberg [13] (n = 11)	Supine, flat couch, knee support. No bowel or bladder instructions.	0.82	1.25	1.02	3 implanted Calypso^® ^markers. Real time tracking of transponder position for 8 minutes, to provide information about intra-fraction motion. "Average" CTV to PTV margins, calculated using the method of van Herk [17], to give 90% probability of covering the target with at least 95% of the prescribed dose.

Stroom [16] a) Supine (n = 15)	Supine. Knee roll, foot support Suppository prior to planning CT; partially full bladder for all CTs	0.40	0.82	0.83	CT scan in treatment position, repeated at weeks 2, 4 and 6 of treatment. Position of prostate registered with initial treatment planning CT. CTV to PTV margins required to cover target with an unspecified isodose line are calculated using the formula: CTV-PTV = 2Σ_tot _+ 0.7σ_tot_, where Σ_tot _and σ_tot _are the quadratically summed contributions of translational set-up uncertainty and internal organ motion.

Stroom [16] b) Prone (n = 15)	Prone. Belly board. Otherwise as above	0.37	0.66	0.88	As above.

Poli [18] (n = 387)	Supine, foam between knees, ankles in Styrofoam block. Full bladder, no bowel instructions.	0.77 right 0.66 left	1.11 sup 0.69 inf	0.27 ant 1.49 post	Daily localization of target using 2D BAT ultrasound for at least 4 consecutive fractions (average 27 per patient). Margins required for 95% probability of target coverage, including the effect of systematic shift (average 0.61 cm posteriorly).

Tinger [19] (n = 8)	Supine, alpha cradle. Urethrogram, rectal probe. Full bladder. No bowel instructions.	0.53	0.73	0.66	Weekly CT, registered to planning CT, to measure center of volume motion of the prostate. Daily EPIs registered to simulator films to measure setup displacement. Data were provided on standard deviation (SD) of total uncertainty of CTV position, from which we calculated margins required to give 95% probability of CTV coverage (CTV-PTV margin calculated as SD × 1.65).

Meijer [20] (n = 30)	Position and immobilization not specified. Bladder instructions given. Bowel instructions not specified.	0.40	0.80 sup 1.10 inf	0.80 ant 1.10 post	4 fiducial markers. Simulation study based on 8 CT scans spaced over the course of treatment. Set-up to skin markers then daily on-line imaging, with no correction of set-up errors. Margins calculated using a dose warping technique to give 90% probability of covering the CTV with at least 95% of the prescribed dose.

Beltran [21] (n = 40)	Position, immobilization, bladder and bowel instructions not specified.	0.73	0.81	1.05	4 fiducial markers. Set up to skin markers, then daily imaging without correction of set-up errors. Margins were calculated using the method of van Herk [18], to give 90% probability of covering the CTV with at least 95% of the prescribed dose.

Nairz [22] (n = 27)	Supine, immobilization not specified, no bowel or bladder instructions	0.87	1.20	1.58	Daily cone beam CT without correction of set-up errors. Margins were calculated using the method of van Herk [17], to give 90% probability of covering the CTV with at least 95% of the prescribed dose.

Graf [23] (n = 23)	Supine, no rigid immobilization. Full bladder, no bowel instructions (although scan repeated if excessive rectal filling)	0.70	0.95	0.95	3 - 5 fiducial markers. Daily EPI without corrections. Margins were calculated using the method of Van Herk [17].

*Abbreviations*: As in table 3. Also, 2D = 2-dimensional; SD = standard deviation; σ_tot _= total random variation; Σ_tot _= total systematic variation.

**Table 5:** CTV to PTV margin recommendations in various series, with image guidance

Series (number of patients)	Treatment set-up details	CTV - PTV margin requirement (cm)			Comments
			
		R-L	S-I	A-P	
Present series (n = 46)	As in table 4	0.36	0.37	0.37	As in table 4, with correction of isocenter placement errors 3 mm or greater in size on R-L and S-I axes, 2 mm or greater on A-P axis. No post-correction EPI.

van der Heide [5] (n = 453)	Supine, knee cushion. Empty bladder, no bowel instructions.	0.18	0.25	0.40	2 - 4 fiducial markers. Daily aSi EPI. Correction of all errors prior to treatment. Standard deviations were provided, from which we calculated margins required to give 95% probability of CTV coverage (CTV - PTV margin calculated as SD × 1.65 [6]).

Cheung [7] (n = 33)	Supine, vacuum lock bag. Empty bladder and rectum.	0.30	0.30	0.40	3 fiducial markers. Pre- and post-RT EPI days 1-9 to calculate individualized CTV-PTV margins (averages shown), which were used during the IMRT boost phase, during which daily on-line correction was performed according to fiducial marker position. A 2 mm factor was added in quadrature to the total error, to account for inaccuracies in the on-line correction process. Average individualized CTV to PTV margins are shown, although several patients had margins larger than 0.7 cm along the A-P axis.

J Wu [10] (n = 13)	Supine, alpha cradle, soft foam support for lower legs. Empty rectum and partially full bladder (drink 500 mL water 45 mins before) for CT and treatment	ns	0.53	0.60	3 fiducial markers. Daily pre-treatment portal images 3× per week over the course of treatment. CTV to PTV margin required to give 99% probability of CTV coverage by 95% isodose line. Margins calculated according to movement of center of mass.

Litzenberg [13] (n = 11)	Supine, flat couch, knee support. No bowel or bladder instructions.	0.18	0.70	0.58	As in table 4, with the inclusion of intra-fraction motion.

Meijer [20] (n = 30)	As in table 4	0.20	0.40 sup 0.60 inf	0.20	4 fiducial markers. Simulation study based on 8 CT scans spaced over the course of treatment. Set-up to skin markers then daily on-line imaging, with correction of all set-up errors. Margins calculated using a dose warping technique to give 90% probability of covering the CTV with at least 95% of the prescribed dose.

Beltran [21] (n = 40)	As in table 4	0.43	0.49	0.48	As in table 4, with daily correction of all errors.

Nairz [22] (n = 27)	As in table 4	0.61	0.96	1.07	As in table 4, with daily correction of all errors.

Graf [23] (n = 23)	As in table 4	0.49	0.51	0.48	As in table 4, with daily correction of all errors.

Q Wu [24] (n = 28)	Not stated	0.30	0.30	0.30	15 CT scans obtained during the course of treatment and registered with respect to bony anatomy with planning CT. Evaluation of both image-based and contour-based registration methods. Analysis based on both geometric and dosimetric parameters. Estimated CTV to PTV margins required to allow a dose reduction on the prostate (D99) of not more than 2% for 90% of patients.

*Abbreviations*: As in table 4.

Our indirect method of estimating intra-fraction motion, because it is based on the comparison of prostate position on only two EPIs, may be less accurate than methods which involve real-time tracking of the prostate's position over the course of treatment [12,13]. It also may not capture spontaneous target displacements due to physiologic or physical factors (e.g. bowel gas or patient movement). Nevertheless, our results are not dissimilar to other published reports which used different methods. This includes along the S-I axis, even though the 3 mm CT slice thickness theoretically introduces an additional error of +/- 1.5 mm (one half the slice thickness) compared to other axes. An exception is along the L-R axis, where our estimate of SD was larger than what was reported in other studies that provided this information. There are a number of possible explanations for this observation. There is some subjectivity inherent to our matching procedure, such that inter and intra-observer variability in determination of isocenter placement errors is likely to be on the order of 1 -- 2 mm. Corrections were performed manually, by entering the treatment room and moving the couch in the direction(s) opposite to the error. Accuracy of the digital readout on the treatment couch was to ± 1 mm, and accuracy of the manual correction process was likely similar to this. Post-correction EPIs were not performed, which would have confirmed the correct couch adjustments but at a cost of introducing extra time and radiation exposure. It is apparent that some "corrections" were performed in the wrong direction, resulting in a potentially much larger isocenter placement error than existed on the pre-treatment EPI. Although we are not able to identify with certainty all of the individual treatment fractions for which this occurred, we know this is the explanation for at least some of the outlying points on figure 3, as mentioned previously. We did not exclude from the analysis any data points that we felt had been "corrected" in the wrong direction. Since our procedure is potentially affected by human error, we did not feel the effects of those errors should be omitted from the results. If we had excluded the single point estimate of IFM of 2.6 cm to the left (figure 3b), the standard deviation of IFM along the L-R axis would have fallen from 0.19 cm to 0.15 cm, and the CTV to PTV margin required to give a 95% likelihood of CTV coverage along that axis would have decreased by 0.06 cm. Although this additional margin reduction is perhaps trivial, the case for automated correction of errors is strong especially with hypofractionated RT, since a geographic miss on even one out of 16 fractions could result in a significant lowering of tumor control probability.

Tables 4 and 5 respectively shows estimates of required CTV to PTV margins from a selection of studies without [5,13,16-23] and with [5,7,10,13,20-24] the use of image guidance. As with the quantification of intra-fraction motion, a variety of different techniques have been used to estimate margin requirements, and the level of confidence of target coverage with the specified margins varies between different reports, making direct comparisons difficult. What can be concluded, however, is that the use of image guidance techniques permits the use of narrower CTV to PTV margins than if these techniques are not used. While our estimates of CTV to PTV margin requirements along the S -- I and A -- P axes are comparable to other reports, our estimate of margin requirement along the L -- R axis appears to be slightly larger than in the other reports using image guidance. This is related to our larger estimate of intra-fraction motion along this axis, for reasons outlined in the previous paragraph. Since margins along the L-R axis have the least effect on treatment morbidity, there is probably little to be gained from a method that provides more precise estimates of IFM.

Our estimates of intrafraction motion, and therefore of CTV to PTV margin requirements, are based on a single pair of orthogonal during-treatment EPIs for each fraction, which were compared with a corresponding pair of pre-treatment EPIs. This might under or overestimate the true extent of intra-fraction motion. The use of electromagnetic transponders [13] and cine-MRI imaging [14] have shown that the prostate can move throughout the course of a single radiation treatment. If either or both of the pair of EPIs happened to capture a transient extreme in position of the prostate, this might lead to incorrect conclusions about the required size of the CTV to PTV margins, at least if this happened in a systematic way. Whether or not the estimated CTV to PTV margin requirements in figures 1 and 2 (without and with image guidance) are accurate, however, the relative reductions in PTV margins that are possible with our image guidance protocol are likely to be real, since under or overestimation of intra-fraction motion should be a random process and should therefore occur similarly whether or not image guidance is being used.

## Conclusions

In the radiotherapy of localized prostate cancer, an image guidance strategy using implanted fiducial markers, daily pre-treatment portal imaging, and adjustment of isocenter position based on pre-defined criteria, permits the use of narrower CTV to PTV margins, and a smaller PTV volume, without compromising coverage of the target. The CTV to PTV margins used in this study (1.0 cm along all axes except 0.5 cm posteriorly) provided reliable coverage of the target with maximum sparing of the rectum. Although the anterior, L-R and S-I CTV to PTV margins of 1.0 cm appear over-generous, they may be justifiable to account for contouring uncertainty and/or microscopic disease extension. Any strategy that permits the use of narrower CTV to PTV margins may allow for safe dose escalation, which may improve the outcome of radical RT for prostate cancer.

## Competing interests

The authors declare that they have no competing interests.

## Authors' contributions

JW designed the study, with assistance from DS and RP. PC analyzed the data. All authors helped to interpret the findings. DS wrote the manuscript, which was approved by all authors.

## References

[B1] Zietman A DeSilvio M Slater J Rossi C Miller D Addams J Shipley W Comparision of conventional-dose vs high-dose conformal radiation therapy in clinically localized adenocarcinoma of the prostate: a randomized clinical trial JAMA 2005 294 1233 1239 10.1001/jama.294.10.1233 16160131

[B2] Peeters S Heemsbergen W Koper P van Putten W Slot A Dielwart M Bonfrer J Incrocci L Lebesque J Dose-response in radiotherapy for localized prostate cancer: results of the Dutch multicenter randomized phase III trial comparing 68 Gy of radiotherapy with 78 Gy J Oncol Pract 2006 24 1990 1996 10.1200/JCO.2005.05.2530 16648499

[B3] Kuban D Tucker S Dong L Starkschall G Huang E Cheung M Lee A Pollack A Long-term results of the M.D. Anderson randomized dose-escalation trial for prostate cancer Int J Radiat Oncol Biol Phys 2008 70 67 74 17765406 10.1016/j.ijrobp.2007.06.054

[B4] Skwarchuk M Jackson A Zelefsky M Venkatraman E Cowen D Levegrun S Burman C Fuks Z Leibel S Ling C Late rectal toxicity after conformal radiotherapy of prostate cancer (I): multivariate analysis and dose-response Int J Radiat Oncol Biol Phys 2000 47 103 113 10758311 10.1016/s0360-3016(99)00560-x

[B5] van der Heide U Kotte A Dehnad H Hofman P Lagenijk J van Vulpen M Analysis of fiducial marker-based position verification in the external beam radiotherapy of patients with prostate cancer Radiother Oncol 2007 82 38 45 10.1016/j.radonc.2006.11.002 17141903

[B6] Antolak J Rosen I Childress C Zagars G Pollack A Prostate target volume variations during a course of radiotherapy Int J Radiat Oncol Biol Phys 1998 42 661 672 9806528 10.1016/s0360-3016(98)00248-x

[B7] Cheung P Sixel K Morton G Loblaw D Tirona R Pang G Choo R Szumacher E DeBoer G Pignol J-P Individualized PTV for intrafraction motion during hypofractionated IMRT boost for prostate cancer Int J Radiat Oncol Biol Phys 2005 62 418 425 15890583 10.1016/j.ijrobp.2004.09.051

[B8] Aubry J-F Beaulieu L Girouard L-M Aubin S Tremblay D Laverdière J Vigneault E Measurements of intrafraction motion and interfraction and intrafraction rotation of prostate by three-dimensional analysis of daily portal imaging with radiopaque markers Int J Radiat Oncol Biol Phys 2004 60 30 39 15337537 10.1016/j.ijrobp.2004.02.045

[B9] Chung P Haycocks T Brown T Cambridge Z Kelly V Alasti H Jaffray D Catton C On-line aSi portal imaging of implanted fiducial markers for the reduction of interfraction error during conformal radiotherapy of prostate carcinoma Int J Radiat Oncol Biol Phys 2004 60 329 334 10.1016/j.ijrobp.2004.07.150 15337572

[B10] Wu J Haycocks T Alasti H Ottewell G Middlemiss N Abdolell M Warde P Toi A Catton C Positioning errors and prostate motion during conformal prostate radiotherapy using on-line isocenter set-up verification and implanted prostate markers Radiother Oncol 2001 61 127 133 10.1016/S0167-8140(01)00452-2 11690677

[B11] Letourneau D Martinez A Lockman D Yan D Vargas C Ivaldi G Wong J Assessment of residual error for online cone-beam CT-guided treatment of prostate cancer patients Int J Radiat Oncol Biol Phys 2005 62 1239 1246 15913917 10.1016/j.ijrobp.2005.03.035

[B12] Nederveen A Van Der Heide U Dehnad H Van Moorselaar R Hofman P Lagenduk J Measurements and clinical consequences of prostate motion during a radiotherapy fraction Int J Radiat Oncol Biol Phys 2002 53 206 214 12007961 10.1016/s0360-3016(01)02823-1

[B13] Litzenberg D Balter J Hadley S Sandler H Willoughby T Kupelian P Levine L Influence of intrafraction motion on margins for prostate RT Int J Radiat Oncol Biol Phys 2006 65 548 553 16545919 10.1016/j.ijrobp.2005.12.033

[B14] Ghilezan M Jaffray D Siewerdsen J Van Herk M Shetty A Sharpe M Jafri S Vicini F Matter R Brabbins D Martinez A Prostate gland motion assessed with cine-magnetic resonance imaging (cine-MRI) Int J Radiat Oncol Biol Phys 2005 62 406 417 15890582 10.1016/j.ijrobp.2003.10.017

[B15] Huang E Dong L Chandra A Kuban D Rosen I Evans A Pollack A Intrafraction prostate motion during IMRT for prostate cancer Int J Radiat Oncol Biol Phys 2002 53 261 268 10.1016/S0360-3016(02)02878-X 12023128

[B16] Stroom J Koper P Korevaar G van Os M Janssen M de Boer H Levandag P Heijmen B Internal organ motion in prostate cancer patients treated in prone and supine position Radiother Oncol 1999 51 237 248 10.1016/S0167-8140(99)00061-4 10435819

[B17] Van Herk M Remeijer P Rasch C Lebesque J The probability of correct target dosage: dose-population histograms for deriving treatment margins in radiotherapy Int J Radiat Oncol Biol Phys 2000 47 1121 1135 10863086 10.1016/s0360-3016(00)00518-6

[B18] Poli M Parker W Patrocinio H Souhami L Shenouda G Campos L Podgorsak E An assessment of PTV margin definitions for patients undergoing conformal 3D external beam radiation therapy for prostate cancer based on an analysis of 10,327 pretreatment daily ultrasound localizations Int J Radiat Oncol Biol Phys 2007 67 1430 1437 17208385 10.1016/j.ijrobp.2006.11.004

[B19] Tinger A Michalski JM Cheng A Low D Zhu R Bosch W Purdy J Perez C A critical evaluation of the planning target volume for 3-D conformal radiotherapy of prostate cancer Int J Radiat Oncol Biol Phys 1998 42 213 221 9747840 10.1016/s0360-3016(98)00189-8

[B20] Meijer G de Klerk J Bzdusek K van den Berg H Janssen R Kaus M Rodrigus P van der Toorn P-P What CTV-to-PTV margins should be applied for prostate irradiation? Four-dimensional quantitative assessment using model-based deformable image registration techniques Int J Radiation Oncology Biol Phys 2008 72 1416 25 10.1016/j.ijrobp.2008.03.005 18439767

[B21] Beltran C Herman M Davis B Planning target margin calculations for prostate radiotherapy based on intrafraction and interfraction motion using four localization methods Int J Radiation Oncology Biol Phys 2008 70 289 95 10.1016/j.ijrobp.2007.08.040 17919837

[B22] Nairz O Merz F Deutschmann H Kopp P Schöller H Zehentmayr F Wurstbauer K Kametriser G Sedlmayer F A strategy for the use of image-guided radiotherapy (IGRT) on linear accelerators and its impact on treatment margins for prostate cancer patients Strahlenther Onkol 2008 184 663 7 10.1007/s00066-008-1874-7 19107347

[B23] Graf R Wust P Budach V Boehmer D Potentials of on-line repositioning based on implanted fiducial markers and electronic portal imaging in prostate cancer radiotherapy Radiation Oncology 2009 4 13 10.1186/1748-717X-4-13 19397824 PMC2683853

[B24] Wu Q Ivaldi G Liang J Lockman D Yan D Martinez A Geometric and dosimetric evaluations of an online image-guidance strategy for 3D-CRT of prostate cancer Int J Radiat Oncol Biol Phys 2006 64 1596 1609 16580509 10.1016/j.ijrobp.2005.12.029

